# Paraxanthine Supplementation Increases Muscle Mass, Strength, and Endurance in Mice

**DOI:** 10.3390/nu14040893

**Published:** 2022-02-20

**Authors:** Ralf Jäger, Martin Purpura, Shawn D. Wells, Kylin Liao, Ashok Godavarthi

**Affiliations:** 1 Ingenious Ingredients L.P., Lewisville, TX 75056, USA; martin.purpura@increnovo.com (M.P.); shawn@ingeniousingredients.com (S.D.W.); kylin@ingeniousingredients.com (K.L.); 2 Increnovo LLC, Milwaukee, WI 53202, USA; 3 Radiant Research Services Pvt. Ltd., Bangalore 560058, India; ashok@radiantresearch.in

**Keywords:** sports performance, nootropics, ergogenic aid, muscle mass, cardiovascular health

## Abstract

Paraxanthine is a natural dietary ingredient and the main metabolite of caffeine in humans. Compared to caffeine, paraxanthine exhibits lower toxicity, lesser anxiogenic properties, stronger locomotor activating effects, greater wake promoting properties, and stronger dopaminergic effects. The purpose of this study was to evaluate the potential beneficial effects of paraxanthine supplementation on muscle mass, strength, and endurance performance in comparison to the control and other ingredients commonly used by athletes: L-theanine, alpha-GPC, and taurine. Male Swiss Albino mice from five groups (*n* = 8 per group) were orally administered paraxanthine (20.5 mg/kg/day, human equivalence dose (HED) 100 mg), L-theanine (10.28 mg/kg/day, HED 50 mg), alpha-GPC (41.09 mg/kg/day, HED 200 mg), taurine (102.75 mg/kg/day, HED 500 mg), or control (carboxy methyl cellulose) for 4 weeks. Exercise performance was evaluated using forelimb grip strength and treadmill endurance exercise. All animals were subject to treadmill training for 60 min 5 days per week. Blood draws were utilized to analyze lipid profile, liver health, renal function, and nitric oxide levels. Paraxanthine significantly increased forelimb grip strength by 17% (*p* < 0.001), treadmill exercise performance by 39% (*p* < 0.001), gastrocnemius and soleus muscle mass by 14% and 41% respectively (both *p* < 0.001), and nitric oxide levels by 100% compared to control (*p* < 0.001), while reducing triglyceride (*p* < 0.001), total cholesterol (*p* < 0.001), LDL (*p* < 0.05), and increasing HDL (*p* < 0.001) compared to control, and compared to L-theanine, alpha-GPC, and taurine. Results from this initial investigation indicate that, when compared to the control, L-theanine, alpha-GPC, and taurine, paraxanthine is an effective ingredient for various aspects of sports performance and may enhance cardiovascular health.

## 1. Introduction

Paraxanthine (1,7-dimethylxanthine) is a natural dietary ingredient and the main metabolite of caffeine in humans [1]. Supplementation with caffeine in humans increases various aspects of athletic performance, including muscular strength, muscular endurance, and aerobic endurance [2]. Several mechanisms of action have been proposed to explain the ergogenic properties of caffeine, including enhanced free fatty acid oxidation resulting in glycogen sparing and adenosine receptor antagonism [3]. Paraxanthine has been linked to increased lipolysis after caffeine ingestion in humans [4]. The activation of the central nervous system has been widely accepted as the primary mechanism by which caffeine increases performance [2]. Caffeine induces locomotor activation by its ability to block adenosine receptors [3]. Interestingly and when compared to caffeine, paraxanthine in equine forebrain exhibits higher binding potencies for adenosine A1 and A2A receptors [5] and, in rats, has a stronger locomotor activating effect [6] leading paraxanthine to also function as a central nervous stimulant in a manner similar to caffeine.

In addition to being an adenosine receptor antagonist, paraxanthine, and not caffeine, has been shown to potentiate nitric oxide (NO) neurotransmission [7]. NO is produced in the brain by GABAergic interneurons and induces glutamate release by converting guanosine-3′,5′-monophosphate (GMP) into cyclic GMP (cGMP). The enzyme phosphodiesterase 9 (PDE9) terminates NO-cGMP signaling by metabolizing cGMP back to GMP [7]. PDE9 is a specific target of paraxanthine and is not influenced by caffeine, or the other methylxanthine metabolites theophylline and theobromine [7]. In addition, increases in nitric oxide levels have been linked to increases in blood flow secondary to stimulation of NO production in the capillary endothelium while also being linked to changes in aerobic exercise performance and cardiovascular health. Increased blood flow to working muscles can potentially improve sports performance, by increasing work efficiency, modulating force production, and reducing phosphocreatine degradation [8]. Acute administration of caffeine increases nitric oxide production and augments endothelium-dependent vasodilation [9], but this effect has yet to be established with paraxanthine administration. The caffeine metabolite, theobromine, is a vasodilator and increases cerebral and muscular blood flow while theophylline functions as a relaxant in vascular, bronchiole, muscular, and respiratory tissues [10]. Paraxanthine administration has demonstrated the ability to potentiate NO transmission, resulting in increased dopamine release [7]. The dopamine system plays a crucial role in motor physiology by directly controlling motoneuron function [11]. Currently, the extent to which paraxanthine supplementation increases nitric oxide levels is unknown. Thus, it remains possible that potential ergogenic outcomes associated with paraxanthine, if realized, could be due to enhancements in NO production and signaling.

Many dietary ingredients have been studied for their abilities to promote skeletal muscle hypertrophy, strength, and endurance performance [9], including alpha-glycerophosphocholine (alpha-GPC), L-theanine, and taurine. Cholinergic nerves trigger muscle contractions during exercise and supplementation with choline has been shown to reduce exercise-induced declines in choline levels and subsequently increase endurance performance [12]. In addition, alpha-GPC supplementation has been shown to increase strength [13] while L-theanine has been shown to improve mental regeneration after exercise [14] and has been linked to the modulation of dopaminergic transmission [15]. Finally, taurine is found in high abundance in skeletal muscle and plays a wide variety of physiological functions [9]. A recent meta-analysis of 10 human clinical studies of oral taurine supplementation concluded that varying doses of taurine, administered acutely or chronically, can improve endurance performance [16].

The potential ergogenic effects of paraxanthine as well as its ability to impact nitric oxide levels are currently unknown. The purpose of this study was to evaluate the potential beneficial effects of paraxanthine supplementation in mice on muscle mass, muscular strength and endurance, and nitric oxide levels in comparison to control, alpha-GPC, taurine, and L-theanine. Two primary hypotheses exist within this investigation. First, it is hypothesized that paraxanthine administration will lead to improvements in strength and endurance performance when compared to control, taurine, alpha-GPC, and theanine. Second, it is hypothesized that, when compared to control, paraxanthine will increase nitric oxide production. Finally, limited data exists to better understand the tolerability of paraxanthine administration from a health and adverse event perspective and as a result this study will seek to better understanding the impact paraxanthine has on kidney, liver, and heart tissues as well as commonly clinical markers of safety and health.

## 2. Methods

### 2.1. Animals and Experiment Design

Forty 8-week-old male Swiss Albino mice were housed in an animal room at a constant temperature (22 ± 3 °C) and humidity (30–70%) under a 12:12 h light-dark cycle with standard laboratory diet (Purina 5L79, Rat and Mouse 18% protein; PMI Nutrition International, Brentwood, MO, USA). Distilled water was provided ad libitum. All animal experiments were reviewed and approved by the Institutional Animal Ethical Committee (IAEC) of Radiant Research Services Pvt. Ltd. (Bangalore, India). All research was conducted in accordance with the guidelines of the committee for the purpose of control and supervision of experiments on animals (CPCSEA Registration Number-1803/PO/RcBi/S/2015/CPCSEA).

After one week of acclimation, the animals were randomly divided by body weight into five groups (*n* = 8 per group in each test) for oral treatment once a day, at approximately the same time each day (±1 h), for 28 consecutive days: (1) vehicle control; (2) paraxanthine; (3) L-theanine; (4) alpha-GPC; and (5) taurine. The dose administered to the mice was calculated using US Food and Drug Administration for human equivalence doses (HED), assuming a human weight of 60 kg. The following HED were used in this study: 100 mg paraxanthine, (ENFINITY™, Ingenious Ingredients, L.P Lewisville, TX, USA; mouse dose: 20.5 mg/kg bw/day); 200 mg alpha-GPC mg (YangLing Daily Health Bio-Engineering Technology Co., Ltd., Xi’an, Shaanxi Province, China; mouse dose: 41.09 mg/kg bw), 50 mg L-theanine (Hangzhou Qinyuan Natural Plant High-tech Co., Ltd. Hangzhou, Zhejang Province, China; mouse dose: 10.28 mg/kg bw), and 500 mg taurine (Jiangyin Huachang Food Additive Co., Ltd., Jiangyin, Jiangsu Province, China; mouse dose: 102.75 mg/kg bw). A total of 0.5% Carboxy Methyl Cellulose sodium was used as vehicle and the test item formulations were prepared daily. Dosing was conducted via oral gavage using disposable polypropylene syringes with sterilized stainless steel gavage tubes. Food intake was monitored daily while water intake was ad libitum. BW was recorded weekly. Animals were not trained on day 28, with all animals receiving their final dose on day 28 given 1 h prior to completing strength and endurance testing. Animals were then kept overnight for fasting and on day 29 the animals were sacrificed for final collection of blood and tissue samples. Thus, all collected blood and tissue samples were deemed to be collected in a basal state. An overview of the research design can be found in Table 1.

### 2.2. Sample Collection

Animals were not trained on day 28 and were kept for overnight fasting. All animals were euthanized by 95% CO_2_ after 28 full days of following their assigned treatment. Blood was collected immediately after euthanization by the retro-orbital route and was immediately centrifuged at 1500× *g* for 10 min at 4 °C before having all serum transferred into cryogenic tubes and stored at −80 °C. The liver, heart, gastrocnemius, and soleus were excised and weighed.

### 2.3. Forelimb Grip Strength Test

The forelimb grip strength was measured on days 0 and 28 by using a stainless-steel grill to assess muscle strength (Orchid Scientific and Innovative India Pvt Ltd., Nashik, India). Grip strength was measured 1 h after treatment. Briefly, each mouse was first placed in the testing room for 10 min to acclimate. Each mouse was then placed over the top of the grid of a grip-strength meter to allow the mouse to grasp the grid with all four paws. The mouse was held by the base of the tail without pressing down upon the grid. The animal was then gently pulled backwards away from the grid by the tail pulling along the axis of the grip strength measurement. The speed was slow enough to let the mouse develop a resistance against the pulling force and the score that is displayed (gf) on the screen of the grip strength measurement was recorded once the mouse released the grid. Each animal performed three independent trials and the mean of the three trials was calculated and recorded [17]. 

### 2.4. Exercise Training

During the treatment period, exercise training was completed using a motorized treadmill (Exer 3/6, Columbus Instruments international, Columbus, OH, USA) at a moderate intensity of 20 cm/sec as maximal running speed, an incline of 10 degrees and a shock intensity of 0.2 mA, for 10 min. The speed of the treadmill was manually adjusted by increasing the belt speed by 5 cm/sec every 2 min throughout the total duration of 10 min. All animals were adapted to this procedure daily 60 min after dosing for 5 days in a week during the treatment period.

### 2.5. Treadmill Endurance Test

On the 28th day of each respective treatment, all animals were subjected to a muscle endurance test. Muscle endurance was accomplished on a motorized treadmill using speeds that ranged from 5 to 50 cm/s, and an incline of 10 degrees. Uphill running involves concentric muscle contractions and increases the muscular work compared to running on a flat surface resulting in faster exhaustion. Per the methods of Castro et al. [17], the belt speed started at approximately 15 cm/s and increased by 5 cm/s every 2 min until it reached a speed of 50 cm/s. Animals were subjected to the treadmill test until exhaustion. The points of exhaustion were defined at the time point at which the animals fell down into the shock zone. The distance traveled (cm) was measured as a marker of exercise performance. 

### 2.6. Clinical Biochemical Profiles 

After 28 days of treatment, the collected serum was analyzed for clinical biochemical variables including aspartate aminotransaminase (AST), alanine aminotransaminase (ALT), alkaline phosphatase (ALP), uric acid (UA), creatinine, total cholesterol (TC), triglycerides (TG), high-density lipoproteins (HDL), and low-density lipoproteins (LDL) were measured using an auto analyzer (EM360, ERBA Diagnostics Mannheim GmbH, Mannheim, Germany). Nitric oxide was analyzed using standard ELISA assay kits (Lot No: E-BC-K035-M, Elabscience, Houston, TX, USA).

### 2.7. Statistical Analysis

Data are presented as mean ± standard deviation (SD). Baseline and group differences were assessed using one-way ANOVA. Changes across time were assessed between groups by first calculating the change from baseline (delta) score for all endpoints before using a one-way ANOVA to assess between-group differences. Because all comparisons against paraxanthine were of primary interest in this study, the Dunnett’s pairwise comparison test was applied when a statistically significant differences was found between groups. In addition, Bonferroni corrections were applied in instances where comparisons between others groups were considered. Data was captured into Microsoft Excel (Seattle, WA, USA) and analyzed using SPSS v23. GraphPad PRISM Software, Version 5.01 (GraphPad Software, Inc., San Diego, CA, USA) was used for presentation of data in the figures. The level of statistical significance was set at *p* < 0.05.

## 3. Results

### 3.1. Effect on Body Weight and Feed Consumption

Body weight and feed consumption of each group of animals were assessed weekly and averaged across the entire 4-week study. Feed intake was not different between groups at week 1 (*p* = 0.052), week 2 (*p* = 0.30), week 3 (*p* = 0.10), or week 4 (*p* = 0.10) nor was the average feed intake across the entire study (*p* = 0.08). Body mass between groups was not different at week 0 (*p* = 0.14), week 1 (*p* = 0.73), week 2 (*p* = 0.91), week 3 (*p* = 0.06) Feed intake was not different between groups at week 1 (*p* = 0.052), week 2 (*p* = 0.30), or week 3 (*p* = 0.10) nor was the average recorded body mass (*p* = 0.61). Week 4 body mass was different between the groups (*p* < 0.001). Dunnett’s post-hoc revealed that week 4 paraxanthine body mass was different than theanine (*p* < 0.001), Alpha-GPC (*p* < 0.001), and taurine (*p* = 0.001), but not control (*p* = 0.99). The body weight is summarized in Table 2, and feed consumption in Table 3.

### 3.2. Effect of Supplementation on Forelimb Grip Strength

**Figure 1:**
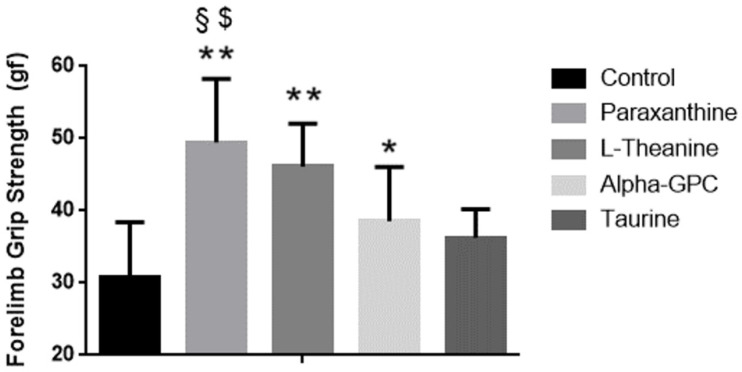
Changes in forelimb grip strength in mice. Data is presented as means ± SD (*n* = 8 in each group). Unit of measures is grams of force. ** = p < 0.001 vs. control, * = p < 0.05 vs. control, § *= p* < 0.05 vs. alpha-GPC, $ = *p* < 0.01 vs. taurine.

Baseline forelimb grip strength in the mice was different between groups (control 88.9 ± 1.8, paraxanthine 90.6 ± 0.9, L-Theanine 87.9 ± 2.6, alpha-GPC 91.0 ± 0.4, taurine 90.9 ± 0.9, *p* < 0.001). Dunnett’s post-hoc tests both indicated that paraxanthine had significantly higher strength levels than theanine at baseline (*p* = 0.004). After 28 days of supplementation, changes in forelimb grip strength were greater in paraxanthine (49.4 ± 8.8) when compared, respectively, to the strength changes observed in control (30.9 ± 7.5, *p* < 0.001), alpha-GPC (38.6 ± 7.5, *p* = 0.013), and taurine (36.3 ± 3.9, *p* = 0.002), but were not different when compared to the strength changes observed in theanine (46.1 ± 5.9, *p* = 0.74) groups (See Figure 1). Respectively, the strength changes observed in paraxanthine were 16.9% greater than control, 8.1% greater than alpha-GPC, and 10.1% greater than theanine. No other groups exhibited significant changes in strength (*p* > 0.05) when compared to the paraxanthine or control groups, see Figure 1. 

### 3.3. Effect of Supplementation on Treadmill Performance

**Figure 2:**
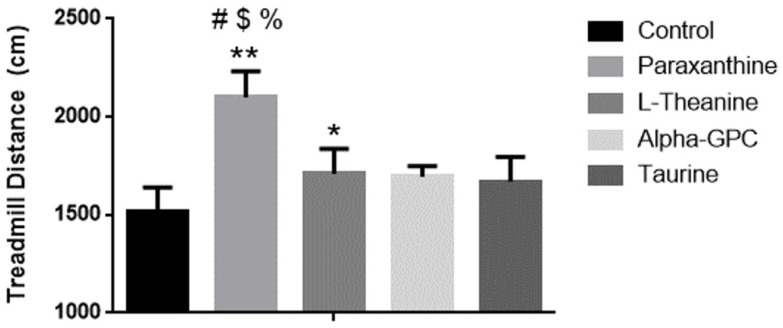
Distance traveled on treadmill in centimeters by mice after 28 days of supplementation. ** = p < 0.001 vs. control, * = p < 0.05 vs. control, # = *p* < 0.001 vs. L-theanine, $ = *p* < 0.001 vs. alpha-GPC, % = *p* < 0.001 vs. taurine. Data is represented by mean ± SD (*n* = 8 in each group).

Baseline treadmill performance was not different between groups (control 267.1 ± 29.3 cm, paraxanthine 255.3 ± 30.7 cm, L-Theanine 265.4 ± 32.9 cm, alpha-GPC 248.6 ± 42.2 cm, taurine 256.9 ± 29.3 cm, *p* = 0.79). Paraxanthine supplementation for 28 days resulted in significantly longer treadmill distances being traveled when compared to control (38.7%, *p* < 0.001), L-theanine (12.9%, *p* < 0.001), alpha-GPC (11.9%, *p* < 0.001), and taurine (10.3%, *p* < 0.001). Moreover, when the changes in treadmill distance were calculated, a significant between-group difference was identified. Post-hoc comparisons revealed that the changes in distance covered by paraxanthine were significantly greater than the change in distance covered by all other groups (*p* < 0.001). L-Theanine supplementation led to significantly greater treadmill distances being traveled when compared to the control (13%, *p* < 0.05). No other changes (*p* > 0.05) in the treadmill distances after 28 days between supplementation groups were realized, see Figure 2.

### 3.4. Effect of Supplementation on Liver Health, Renal Function, Lipid Profiles, and Nitric Oxide

Plasma levels of liver health markers (AST, ALT, and ALP) and renal function (urea and creatinine) were all similar between supplement conditions, see Table 4. Serum levels of triglycerides (*p* < 0.001), total cholesterol (*p* < 0.001), HDL cholesterol (*p* < 0.001), LDL cholesterol (*p* < 0.05), and nitric oxide (*p* < 0.001) were different between conditions. Follow-up post-hoc comparisons revealed that paraxathine had lower levels of triglycerides (82.5 ± 2.7 mg/dL) when compared to control (90.0 ± 2.0 mg/dL, *p* < 0.001), alphaGPC (88.4 ± 3.5 mg/dL, *p* = 0.001), theanine (88.1 ± 3.7 mg/dL, *p* = 0.002), and taurine (90.4 ± 2.5 mg/dL, *p* < 0.001). No other between group changes were identified for triglycerides. Follow-up post-hoc comparisons revealed that paraxathine had lower levels of total cholesterol (87.8 ± 2.3 mg/dL) when compared to control (99.5 ± 1.5 mg/dL, *p* < 0.001), alphaGPC (93.9 ± 4.5 mg/dL, *p* = 0.002), and taurine (96.0 ± 4.3 mg/dL, *p* < 0.001), but were similar to theanine (90.6 ± 2.7 mg/dL, *p* = 0.25). Additionally, when compared to control, theanine (*p* < 0.001) and alpha-GPC (*p* = 0.02) levels were different. Follow-up post-hoc comparisons revealed that paraxathine had higher levels of HDL cholesterol (32.0 ± 0.8 mg/dL) when compared to control (28.0 ± 1.1 mg/dL, *p* < 0.001), taurine (28.4 ± 1.1 mg/dL, *p* < 0.001), alpha-GPC (28.4 ± 1.4 mg/dL, *p* < 0.001), and theanine (28.4 ± 1.1 mg/dL, *p* = 0.002). No other between-group differences were noted for HDL cholesterol. Follow-up post-hoc comparisons revealed that paraxathine had lower levels of LDL cholesterol (52.8 ± 3.9 mg/dL) when compared to control (58.0 ± 1.2 mg/dL, *p* < 0.001) and taurine (56.8 ± 2.2 mg/dL, *p* = 0.03), but were not different between alpha-GPC (56.3 ± 2.3 mg/dL, *p* = 0.07) and theanine (55.3 ± 4.1 mg/dL, *p* = 0.27). No other between-group differences were noted for LDL cholesterol. Nitric oxide levels measured 28 days after supplementation revealed that paraxanthine (13.1 ± 0.6 ng/mL) had significantly higher nitric oxide levels than control (6.5 ± 0.4 ng/mL, *p* < 0.001), theanine (10.2 ± 1.3 ng/mL, *p* < 0.001), alpha-GPC (9.2 ± 2.3 ng/mL, *p* < 0.001), and taurine (7.6 ± 0.3 ng/mL, *p* < 0.001). In comparison to control, theanine (*p* < 0.001) and alpha-GPC (*p* = 0.001) levels of nitric oxide were different. No other between-group differences were reported. Results from all lipid panel markers and nitric oxide can be found in Table 5.

### 3.5. Effect of Supplementation on Muscle Mass and Organ Weight

**Figure 3:**
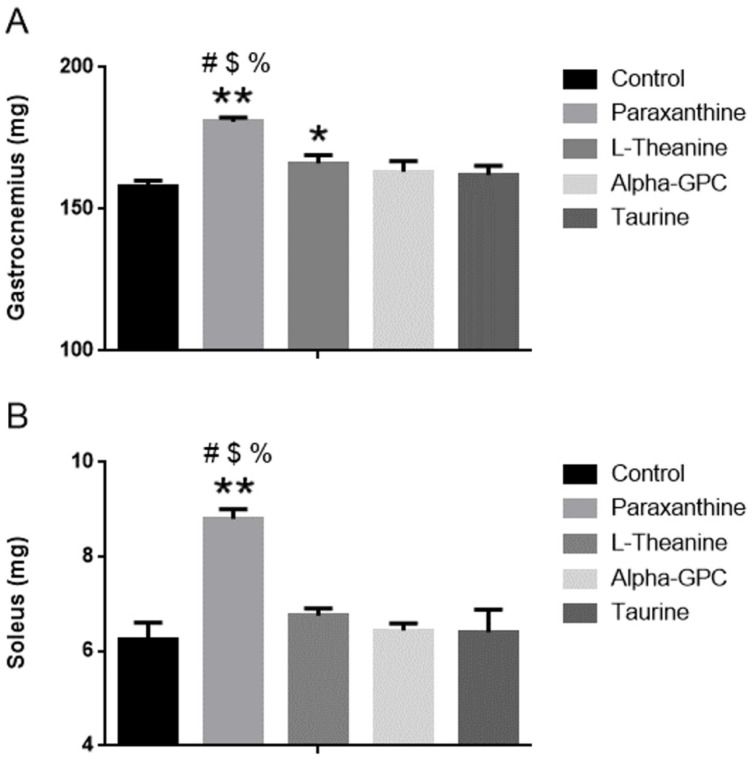
(**A**): Gastrocnemius mass (in milligrams) in mice after 28 days of supplementation. (**B**): Soleus mass (in milligrams) in mice after 28 days of supplementation. * = *p* < 0.05 vs. control, ** = *p* < 0.001 vs. control, # = *p* < 0.001 = vs. L-theanine, $ = *p* < 0.001vs. alpha-GPC (*p* < 0.05), % = *p* < 0.001 vs. Taurine. Data represented by Mean ± SD (*n* = 8 in each group).

As seen in Figure 3A, gastrocnemius mass levels were significantly greater 28 days after paraxanthine supplementation (180.8 ± 1.6 mg) when compared to control (14%, 158.1 ± 1.9 mg, *p* < 0.001), L-theanine (9%, 166.1 ± 2.9 mg, *p* < 0.001), alpha-GPC (11%, 163.1 ± 3.8 mg, *p* < 0.001), and taurine (12%, 162.0 ± 3.3 mg, *p* < 0.001). When compared to control (*p* < 0.05), both L-Theanine and alpha-GPC improved gastrocnemius muscle mass. No other between-group changes were observed after completion of the 28 days supplementation protocol. Soleus mass levels (see Figure 3B) were significantly greater 28 days after paraxanthine supplementation (8.80 ± 0.20 mg) when compared to control (41%, 6.26 ± 0.34 mg, *p* < 0.001), L-theanine (30%, 6.75 ± 0.16 mg, *p* < 0.001), alpha-GPC (37%, 6.44 ± 0.15 mg, *p* < 0.001), and taurine (38%, 6.40 ± 0.48 mg, *p* < 0.001). When compared to control (*p* < 0.05), L-Theanine improved gastrocnemius muscle mass. No other between-group changes in soleus muscle mass were observed after completion of the 28 days supplementation protocol. Gross pathology evaluation of the heart and liver showed no differences between groups for any of the treatments, see Table 6.

## 4. Discussion

The intent of this project was to evaluate, in mice, the impact of ingesting a single acute dose of paraxanthine, L-theanine, alpha-GPC, and taurine for 28 days. Primary outcomes were treadmill distance and forearm strength while secondary outcomes were considered to be changes in nitric oxide levels, health and safety markers, and tissue weights (skeletal muscle and organ). When compared to control, paraxanthine significantly improved treadmill distance, grip strength, muscle mass, and nitric oxide levels while also improving blood lipids. L-theanine moderately increased treadmill distance, forearm strength, nitric oxide and gastrocnemius muscle mass, but not soleus muscle mass level while alpha-GPC significantly increased forearm strength and nitric oxide level when compared to control.

Mechanisms of paraxanthine’s potential ergogenic effects include (A) an increase in plasma free fatty acids, a source of fuel that the body can utilize to produce energy [4], (B) a reduction of plasma K^+^ concentrations which may attenuate the onset of skeletal muscle fatigue, and (C) an increase in calcium ions in the skeletal muscle, which is involved in muscle contractions [18]. While sports nutrition studies of paraxanthine are lacking at this time, previous work has illustrated the ability of acute caffeine supplementation to increase strength [2,19] while ergogenic outcomes related to muscular endurance are somewhat mixed [20,21,22,23]. Paraxanthine is the primary metabolite of caffeine metabolism and has been linked to caffeine’s beneficial effects on athletic performance. Findings from the current study highlighted significant increases in endurance (treadmill running) and muscular strength when compared to the control group. Currently, a deeper mechanistic understanding of these outcomes is unknown. While additional work in paraxanthine is needed, the known ability of caffeine to enhance aerobic endurance is well-established in the literature [2] and this outcome for caffeine is thought to be mediated through its ability to antagonize adenosine receptor interaction, which subsequently promotes alertness, cognitive drive, and motivation towards exercise [2]. Of note, Davenport et al. [24], speculated that the principal mechanism for observed performance improvements of caffeine supplementation in a fatiguing time trial is based on a reduction in perceived exertion mediated by increased paraxanthine levels, outcomes that have been demonstrated in caffeine for over 20 years. A key limitation of our performance findings is our lack of other measures of metabolic relevance during or after the exercise bouts. Nonetheless, more research is needed to explore these potential effects. Finally, an extremely intriguing mechanistic consideration for paraxanthine administration is that it is not subject to the genotypic control via *CYP1A2* allele expression, which has been investigated closely for the past several years [25]. In terms of strength enhancement after paraxanthine administration in the present study, enhanced calcium ion metabolization is likely to be considered the candidate mechanism of action [18], but future research needs to confirm whether the ability of paraxanthine supplementation is also exerting these actions. Finally, a surprising outcome was the statistically significant greater increase in gastrocnemius and soleus muscle mass after paraxanthine supplementation when compared to the changes observed in the control group. While no other work in paraxanthine has addressed these outcomes, increased hypertrophy has not been reported in the caffeine literature. While caffeine is known to signal through adrenergic mechanisms [2] and various sex steroid hormones also signal through these processes [26], more experimental research is needed in humans to fully investigate the potential for paraxanthine supplementation to impact muscle hypertrophy. It seems reasonable to hypothesize that this increased muscle activity would explain at least partially the mechanism for increased muscle mass. The increased hypertrophy is also the most likely mechanism by which the animal increased grip strength. While in this experiment, grip strength was measured in the upper arm, we speculated that muscle hypertrophy also occurred in the upper arm based on the observed increased muscle mass in the hindlimb. 

L-theanine administration led to significant increases in treadmill distance and forearm strength. Neither of these ergogenic outcomes have been reported previously in the literature for theanine. Previous studies using L-theanine in animal models of Parkinson’s reported attenuations of motor deficit and mitochondrial dysfunction [27] while another study suggested L-theanine to facilitate improvements in functional recovery after spinal cord injury in rats [28]. In terms of aerobic exercise performance, Williams et al. [29] reported that adding L-theanine (200 mg) to a sorbet in 18 healthy males did not impact heart rate, blood pressure, or heart rate variability. One study was completed using L-theanine supplementation (150 mg L-theanine extract for six weeks) in national level Polish rowers and reported a decrease in IL-10 concentration and a beneficial effect on the disrupted balance between Th1/Th2 [30]. How these changes related to the outcomes observed in the present study remain to be fully explained and consequently more research is needed using L-theanine to better understand its potential to modulate health and performance. 

Supplementation with alpha-GPC also led to significant increases in strength in the present study. These findings align with previous findings of Bellar et al. [13] who reported significant increases in isometric strength using a mid-thigh pull movement in college-aged males after 6 days of 600 mg/day alpha-GPC supplementation using a randomized, double-blind, crossover approach. A later study by Marcus and investigators [31] reported no changes in upper- and lower-body isometric strength in 48 healthy college-aged males who randomly supplemented with either 500 mg alpha-GPC, 250 mg alpha-GPC, 200 mg caffeine, or a placebo for 7 days. Maximum velocity and maximum mechanical power during countermovement jumps were found to be greater in the 250 mg alpha-GPC group. Additional research needs to be conducted using alpha-GPC to better understand its other ergogenic properties and associated mechanisms of action. 

Higher dose, higher frequency, and/or long duration of taurine supplementation has been linked to increased endurance performance through maintaining blood glucose concentrations during exercise [32]. Our study did not show an enhancement of exercise capacity with acute taurine supplementation, potentially due to the difference in supplementation protocol and the type of exercise (run to exhaustion with gradually increasing speed vs. steady speed). 

Nitric oxide plays a role in the regulation of blood flow, mitochondrial respiration, and muscle contractility [33]. Improved blood flow increases the nutrient delivery to the exercising muscle, and aids in the removal of metabolic byproducts waste. Nitric oxide has a differential impact on the nucleus accumbens, therefore paraxanthine might help with stress-related or reward-related behaviors created by physical performance. Yoo and investigators [34] reported on the first human data involving paraxanthine supplementation, wherein they concluded that acute oral ingestion of a 200 mg dose of paraxanthine may affect short-term memory, reasoning, and response time to cognitive challenges. The outcomes were later confirmed in a dose-response study, confirming that paraxanthine may serve as an effective nootropic nutrient at acute doses as low as 50 mg [35]. Certainly, it remains plausible that the observed increases in nitric oxide could explain some of the observed changes in treadmill endurance performance, as exhibited by the mice in this study. Future research should extend these findings to identify if supplementation results in changes in blood flow and other measures of exercise performance. As nitric oxide availability and vascular functions decline with older age, paraxanthine might be specifically effective in middle-aged and older adults.

No experimental conditions changed the markers of kidney and liver health, nor did they influence liver or heart weight. Feed consumption and body weight exhibited no differences between groups during the duration of the study confirming paraxanthine’s safety [36] and providing preliminary indications that all nutritional adjuncts ingested in this study are safe dietary ingredients at the levels administered. In this respect, paraxanthine administration reduced TG, TC, and LDL while increasing HDL and nitric oxide levels. More research needs to be conducted to better understand the potential impact of paraxanthine supplementation on these health markers. L-theanine administration also decreased triglyceride levels, which operated in contrast to an earlier open-label study in patients with major depressive disorder, which showed an increase in triglycerides and a decrease in HDL after supplementing with 250 mg L-theanine for 8 weeks [37]. While blood creatinine levels are an indicator of whole-body muscle mass, the increase in gastrocnemius and soleus muscle mass in the paraxanthine group did not result in significantly different creatinine levels between groups. A key limitation for the reader to consider is that no baseline values of all clinical markers were assessed, thus it remains possible that the paraxanthine exhibited more favorable outcomes, but due to the animal model imposed, the observed changes are noteworthy and require future follow-up investigations in humans.

Finally, studies with caffeine supplementation have shown substantial variability in outcomes within studies, with 35% of cyclists [38], or 28% of elite or professional athletes [39] showing no benefits or even worsening their performance with caffeine supplementation. Fast metabolizers of caffeine more consistently experience ergogenic outcomes in some [40,41], but not all of the available studies [42,43]. The enzyme cytochrome P450 1A2 is responsible for 95% of all caffeine metabolism, including the demethylation of caffeine to paraxanthine [44]. Individuals with a homogenous A allele of the *CYP1A2* gene tend to produce more cytochrome P450 and consequently metabolize caffeine more quickly [45]. The significant performance differences between subjects might be related to the way individuals metabolize caffeine, and direct supplementation with paraxanthine could eliminate that variability. The strengths of our investigations lie primarily with providing some of the initial data for ingredients such as paraxanthine, L-theanine, and alpha-GPC on various exercise and health outcomes. Limitations of our study was the lack of an equivalent dose of caffeine, varying doses of all ingredients to determine the minimal effective and optimal dose, and mechanistic measures. In addition, our study did not include a sedentary control as all of the animals did exercise training. So, we do not know whether positive effects of paraxanthine might be due to the amplification of exercise-induced adaptation or if paraxanthine is effective even without accompanying exercise training. Well-designed studies are needed to confirm the presented findings in humans. 

## 5. Conclusions

Our results suggest that paraxanthine is an effective ingredient for various aspects of sports performance and may enhance cardiovascular health.

## Figures and Tables

**Table 1:** Overview of research design.

Procedure	Day 1	Day 28
Daily housing of constant temperature and humidity with 12:12 h light-dark cycle	X	X
Daily food consumption assessed with standard laboratory diet	X	X
Ad libitum water	X	X
Oral treatment at same time per day of assigned dose	X	X
Forelimb grip strength	X	X
Treadmill endurance test	X	X
Daily exericse treadmill training for 60 min/day, 5 days/week	X	X
Final dose 1 h prior to testing		X
Euthanized following testing		X
Blood collection and analysis of AST, ALT, uric acid, creatinine, total cholesterol, HDL cholesterol, LDL cholesterol, and nitric oxide		X
Excision and weight of liver, heart, gastrocnemius, and sodium		X

**Table 2:** Effect of test substance on body weight (in grams). Data are mean ± SD for *n* = 8 mice in each group. *** = Different than paraxanthine (*p* < 0.001).

Treatment	Basal	Week 1	Week 2	Week 3	Week 4	Average
Control	22.1 ± 0.7	23.9 ± 0.6	26.9 ± 0.6	29.6 ± 0.6	32.9 ± 0.3	27.1 ± 0.5
Paraxanthine	22.0 ± 0.4	23.9 ± 0.6	27.0 ± 0.4	29.7 ± 0.5	32.8 ± 0.5	27.1 ± 0.3
L-Theanine	22.4 ± 0.5	24.2 ± 0.6	26.8 ± 0.7	29.0 ± 0.6	31.6 ± 0.6 ***	26.8 ± 0.5
Alpha-GPC	22.5 ± 0.4	24.1 ± 0.4	27.1 ± 0.5	29.1 ± 0.5	31.7 ± 0.4 ***	26.9 ± 0.4
Taurine	22.5 ± 0.5	24.1 ± 0.7	27.0 ± 0.8	29.1 ± 0.7	32.3 ± 3.1 ***	27.0 ± 0.3

**Table 3:** Effect of test substance on total feed consumption (in grams). Data are mean ± SD for *n* = 8 mice in each group.

Treatment	Week 1	Week 2	Week 3	Week 4	Average
Control	41.6 ± 0.7	43.7 ± 0.6	45.7 ± 0.7	47.8 ± 0.6	44.2 ± 0.6
Paraxanthine	41.7 ± 0.5	43.9 ± 0.7	46.0 ± 0.6	48.3 ± 0.7	44.4 ± 0.6
L-Theanine	41.3 ± 0.9	43.5 ± 0.7	45.3 ± 0.5	47.6 ± 0.5	43.8 ± 0.5
Alpha-GPC	40.9 ± 0.6	43.3 ± 0.4	45.5 ± 0.4	47.5 ± 0.4	43.8 ± 0.3
Taurine	41.0 ± 0.6	43.2 ± 1.3	45.2 ± 1.2	47.4 ± 1.1	43.7 ± 0.7

**Table 4:** Plasma levels of aspartate aminotransferase (AST), alanine aminotransferase (ALT) alkaline phosphatase (ALP), urea, and creatinine after 28 days of treatment.

Treatment	AST (U/L)	ALT (U/L)	ALP (U/L)	Urea (mg/dL)	Creatinine (mg/dL)
Control	41.1 ± 2.5	25.1 ± 1.1	180.8 ± 3.5	30.1 ± 1.4	0.96 ± 0.13
Paraxanthine	39.5 ± 1.6	23.9 ± 1.0	179.4 ± 2.6	29.1 ± 1.0	0.86 ± 0.11
L-Theanine	40.3 ± 2.4	24.3 ± 1.3	177.1 ± 3.7	28.8 ± 1.3	0.89 ± 0.11
Alpha-GPC	38.9 ± 2.1	23.6 ± 1.5	179.3 ± 4.0	29.4 ± 1.4	0.90 ± 0.16
Taurine	40.1 ± 2.4	25.0 ± 1.1	180.4 ± 5.4	29.4 ± 1.6	0.93 ± 0.14

**Table 5:** Serum levels of triglycerides (TG), total cholesterol (TC), high-density lipoprotein cholesterol (HDL), low-density lipoprotein cholesterol (LDL), and nitric oxide levels after 28 days of treatment.

Treatment	TG (mg/dL)	TC (mg/dL)	HDL (mg/dL)	LDL (mg/dL)	Nitric Oxide (ng/mL)
Control	90.0 ± 2.0	99.5 ± 1.5	28.0 ± 1.1	58.0 ± 1.2	6.5 ± 0.4
Paraxanthine	82.5 ± 2.7 **^$%^	87.8 ± 2.3 **^$%#^	32.0 ± 0.8 **^$&%^	52.8 ± 3.9 *	13.1 ± 0.6 **^$%#^
L-Theanine	88.1 ± 3.7	90.6 ± 2.7	29.1 ± 2.5	55.3 ± 4.1	10.2 ± 1.3
Alpha-GPC	88.4 ± 3.5	93.9 ± 4.5	28.4 ± 1.4	56.3 ± 2.3	9.2 ± 2.3
Taurine	90.4 ± 2.5	96.0 ± 4.3	28.4 ± 1.1	56.8 ± 2.2	7.6 ± 0.3

Data are the mean ± SD for *n* = 8 mice in each group. * *p* < 0.05 vs. control, ** *p* < 0.001 vs. control, $ = *p* < 0.001 vs. alpha-GPC, % = *p* < 0.001 vs. taurine, & = *p* < 0.05 vs. L-theanine, # = *p* < 0.001 vs. L-theanine.

**Table 6:** Liver and heart mass in each group. Data represented by Mean ± SD (*n* = 8 in each group). No changes were noted in all groups.

Treatment	Liver (mg)	Heart (mg)
Control	1874 ± 25	189 ± 1.9
Paraxanthine	1870 ± 31	190 ± 1.4
L-Theanine	1866 ± 43	189 ± 2.3
Alpha-GPC	1869 ± 32	189 ± 1.0
Taurine	1834 ± 52	190 ± 1.2

## Data Availability

Data and/or statistical analyses are available upon request on a case-by-case basis for non-commercial scientific inquiry and/or educational use.
